# Association between monocyte-to-lymphocyte ratio and prostate cancer in the U.S. population: a population-based study

**DOI:** 10.3389/fcell.2024.1372731

**Published:** 2024-04-05

**Authors:** Lanyu Wang, Xiaowan Li, Min Liu, Hongyi Zhou, Jianfeng Shao

**Affiliations:** ^1^ Department of Urology, The Affiliated Wuxi People’s Hospital of Nanjing Medical University, Wuxi People’s Hospital, Wuxi Medical Center, Nanjing Medical University, Wuxi, Jiangsu, China; ^2^ Department of Critical Care Medicine, The Affiliated Wuxi People’s Hospital of Nanjing Medical University, Wuxi People’s Hospital, Wuxi Medical Center, Nanjing Medical University, Wuxi, Jiangsu, China; ^3^ Department of Urology, Wuxi No. 2 People’s Hospital (Jiangnan University Medical Center), Wuxi, Jiangsu, China

**Keywords:** monocyte-to-lymphocyte ratio, prostate cancer, NHANES, cross-sectional study, population-based study

## Abstract

**Introduction:**

Monocyte-to-lymphocyte ratio (MLR) is a convenient and noninvasive inflammatory biomarker, and inflammation has been reported to be associated with prostate cancer (PCa). Our objective was to ascertain any possible correlation between PCa and MLR.

**Methods:**

We utilized data from the 1999–2020 cycles of the National Health and Nutrition Examination Survey (NHANES) regarding MLR and PCa. The independent associations of MLR and other inflammatory biomarkers (platelet-to-lymphocyte ratio (PLR), systemic immune-inflammation index (SII), neutrophil-to-lymphocyte ratio (NLR), system inflammation response index (SIRI), and aggregate index of systemic inflammation (AISI)) with PCa was investigated using weighted multivariate logistic regression and generalized additive models. Receiver operating characteristic (ROC) curves were conducted to evaluate and contrast their diagnostic capabilities.

**Results:**

The analysis we conducted comprised 25,367 persons in total. The mean MLR was 0.31 ± 0.14. The prevalence of PCa was 3.1%. A positive association was found between MLR and PCa (OR = 2.28; 95% CI: 1.44, 3.62). According to the interaction tests, age, body mass index (BMI), hypertension, diabetes, and smoking status did not significantly impact the relationship between MLR and PCa (all *p* for interaction >0.05). ROC analysis showed that MLR had a stronger discriminative ability and accuracy in predicting PCa than other inflammatory biomarkers (NLR, SII, AISI, PLR, and SIRI).

**Conclusion:**

MLR might be better than other inflammatory biomarkers (NLR, SIRI, AISI, PLR, and SII) in predicting PCa. American adults who have elevated levels of MLR, NLR, PLR, SII, and AISI should be aware that they have a greater risk of PCa.

## 1 Introduction

Prostate cancer (PCa) is one of the most prevalent malignant tumors worldwide and the second largest cause of cancer-related deaths in males (Kang et al., 2020). Its incidence is expected to increase annually with the global trend of population aging (Conti et al., 2021). The management and prevention of PCa has become a critical public health issue. Studies have shown that oxidative substances released by inflammatory cells can cause cellular and genetic damage, leading to prostate tumorigenesis (Klein and Silverman, 2008; Caruso et al., 2009). Therefore, inflammation, as a controllable risk factor that is becoming more and more prevalent, should be highly prioritized in preventing the occurrence and progression of PCa.

Many studies have explored the potential mechanisms between inflammatory markers and PCa. Low lymphocyte counts are linked to adverse clinical outcomes, which suggest an inadequate host immune response. This may be because reduced levels of CD4^+^ T cells, compromise tumor suppression and defense (Kitayama et al., 2011). Monocytes can produce reactive oxygen, reactive nitrogen, and other cytokines, which inhibit lymphocyte activation, leading to DNA mutations and tumor progression (Pollard, 2004).

The monocyte-to-lymphocyte ratio (MLR) is a novel inflammatory biomarker that integrates the effects of both inflammatory markers. It has high predictive and prognostic value in various cancers, including hepatocellular carcinoma, gastrointestinal stromal tumor, endometrial cancer, bladder cancer, and breast cancer (Cananzi et al., 2019; Shi et al., 2020; Song et al., 2021; Tiainen et al., 2021; Wang Q. et al., 2022). To our interest, the significance of MLR in PCa has also been confirmed. According to a study by Rundle AG et al., tracking MLR trends may be helpful in assessing the risk of PCa in white men (Rundle et al., 2021). In a cohort research involving 1,740 Korean patients, MLR was associated with PCa detection and an independent predictor of PCa, especially for men with larger prostate volumes (Cho et al., 2021). Another study from China demonstrated the importance of MLR as an independent predictor of PCa (Xu et al., 2021). The latest study showed that MLR was an important potential indicator for predicting PCa patients with significantly elevated prostate-specific antigen (PSA) (Zhu et al., 2023). However, no study has investigated the association between the two in the broader American population.

Consequently, our research aims to investigate the association between MLR and PCa in National Health and Nutrition Examination Survey (NHANES) participants. We reasoned that an elevated prevalence of PCa would be linked to a raised MLR.

## 2 Materials and methods

### 2.1 Study population

**FIGURE 1:**
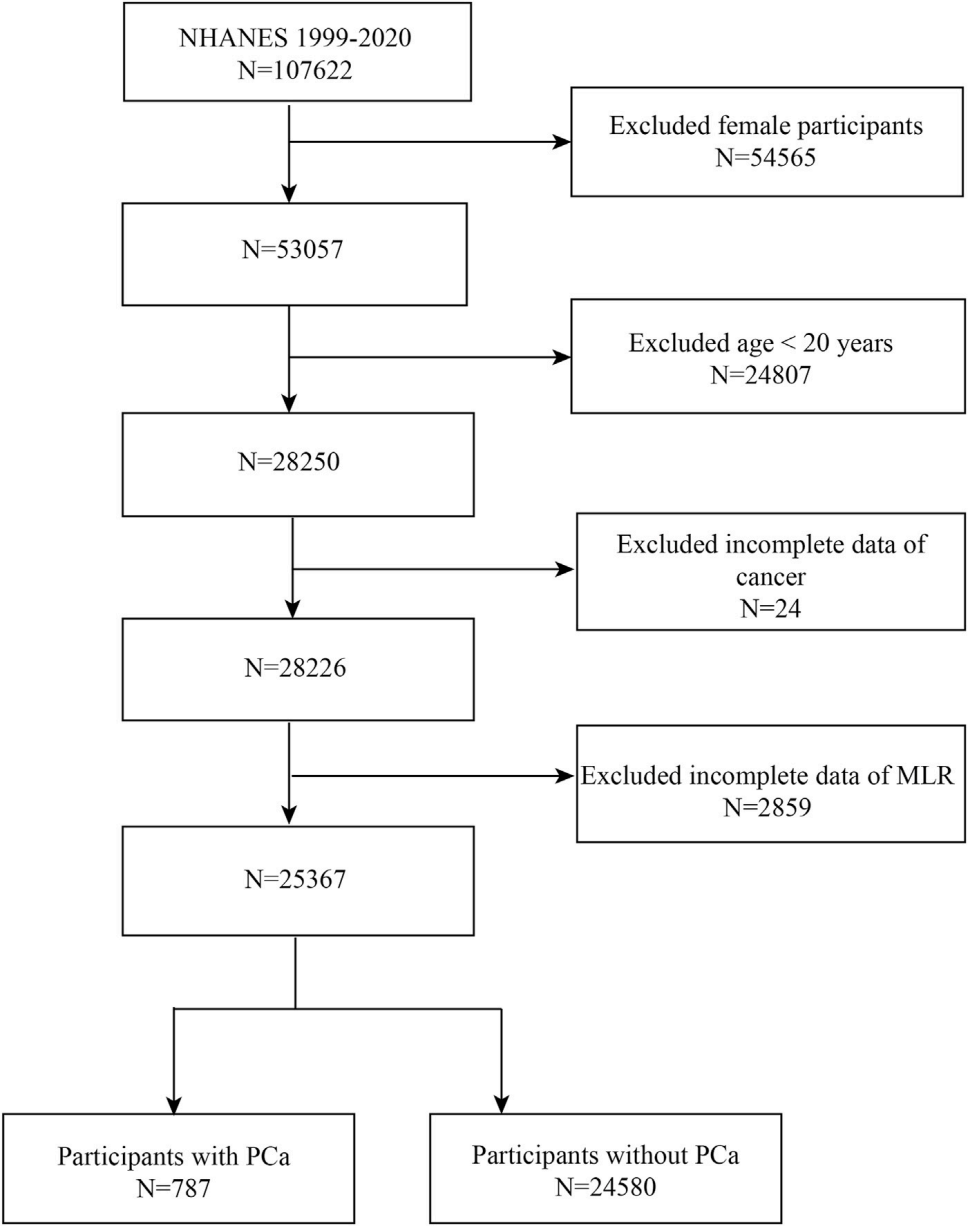
Flowchart of the sample selection from NHANES 1999–2020.

Data were obtained from NHANES between 1999 and 2020. We excluded female (*n* = 54,565) and <20 years old (*n* = 24,807) patients, as well as those with missing data on MLR (*n* = 2,859) and cancer status (*n* = 24), and ended up with 25,367 eligible individuals (Figure 1). Eventually, 24,580 non-PCa participants and 787 PCa patients were enrolled in the research. The people who were enrolled in the study ranged in age from 20 to 85.

### 2.2 Survey description

To gather data regarding the nutritional status and possible health risk factors of US civilian non-institutionalized, NHANES carries out two yearly in this country. This national population-based cross-sectional survey was carried out using a sophisticated stratified, multistage probability cluster sampling design. This study was approved by the Ethics Review Board of the National Center for Health Statistics (NCHS), and informed consent was given by each participant in the NHANES (https://www.cdc.gov/nchs/nhanes/irba98.htm).

### 2.3 Definition of MLR and PCa

Completing a blood count with automated hematology analysis instruments (Beckman Coulter DxH 800 analyzer) allowed for the measurement of the counts of monocytes (MC), lymphocytes (LC), neutrophils (NC), and platelets (PC). The ratio of MC to LC was used to compute the MLR. The systemic immune-inflammation index (SII) was calculated as PC * NC/LC, the system inflammation response index (SIRI) as NC * MC/LC, the platelet-to-lymphocyte ratio (PLR) as PC/LC, and the neutrophil-lymphocyte ratio (NLR) as NC/LC. The history of PCa was the outcome variable of our study, which was evaluated by asking participants if they had ever received a diagnosis of the disease from a physician or another type of medical professional.

To gain more insight into MLR and PCA, we also investigated the relationship between inflammatory markers and PSA. The participants’ serum total PSA was obtained using Beckman Access at the Department of Laboratory Medicine Immunology Division. The serum total PSA concentration (ng/mL) was recorded by the Hybritech PSA technique.

### 2.4 Selection of covariates

A collection of covariates was employed in our investigation to control for plausible confounding variables. These covariates covered a range of various demographic parameters, including age, race, and education level. Additionally, we included a number of anthropometric and laboratory covariates, including smoking status, body mass index (BMI) and alcohol consumption. Furthermore, aspartate aminotransferases (AST), alanine aminotransferases (ALT), triglycerides, serum uric acid, serum total calcium, total cholesterol (TC), eosinophil percentage, basophils percentage, total bilirubin, and family income to poverty ratio (PIR) were among the choices of variables. PIR was divided into three income categories: low (1.3 or less), medium (1.3–3.5), and high (>3.5).

In our analysis, we also accounted for changes in health conditions, such as diabetes and hypertension. We defined hypertension based on the survey question “Ever told you had hypertension,” “taking hypertension prescription,” mean diastolic blood pressure (DBP) ≥ 80 mmHg and/or mean systolic blood pressure (SBP) ≥ 130 mmHg. A three-part criterion was used to define diabetes. The first segment involved self-reported diabetes, and the second segment involved the use of insulin or diabetes medication. The third segment was to determine which patients had diabetes by using glycated hemoglobin or hemoglobin A1c (HbA1c) > 6.5% and fasting plasma glucose (FPG) ≥ 7.0 mmol/L. You can learn more detailed information you need on the webpage of NHANES.

### 2.5 Statistical analysis

We followed the recommendations of the Centers for Disease Control and Prevention (CDC) for statistical analysis, which considered the complex multistage cluster survey and used appropriate NHANES sampling weights. We used percentages to represent categorical variables. The means with standard error (SE) were used to summarize continuous variables. The differences among participants categorized by MLR tertiles were assessed using either a weighted chi-square test (categorical variables) or a weighted Student's t-test (continuous variables). We examined the association between MLR and PCa using multivariable logistic regression in three different models. The covariates in Model 1 were unadjusted. The covariates in Model 2 were adjusted for race, age, and education level. Model 3 was adjusted for age, race, education level, BMI, smoking status, alcohol consumption, TC, triglycerides, total bilirubin, blood uric acid, serum total calcium, eosinophil percentage, basophils percentage, AST, ALT, PIR, diabetes, and hypertension. Additional sensitivity analyses were also performed to evaluate the stability of the results we obtained. Using generalized additive models (GAM) and smooth curve fitting, non-linear relationships were addressed. Additionally, we used log-likelihood ratio tests to compare the segmented regression model (two-segment linear regression model) with the non-segmented (one-line models) in order to find threshold effects. We conducted subgroup analysis by stratifying by age, BMI, hypertension, diabetes, and smoking using stratified multivariable logistic regression models to explore the relationship between MLR and PCa. To evaluate the heterogeneity of the association across subgroups, we used these stratification factors as potential effect modifiers. In addition, we compared the area under the curve (AUC) values and utilized receiver operating characteristic (ROC) curves to assess the predictive ability of inflammatory biomarkers for PCA. To effectively decrease the influence of individual differences and systematic errors, the normalization was performed before ROC analysis. The mode imputation of existing cases was used to input missing values for categorical variables, while the median imputation was used for continuous variables. For all of our statistical analyses, we used R version 4.1.3 and the Empower software suite. *p-*values less than 0.05 indicated statistical significance in the results.

## 3 Results

### 3.1 Participants characteristics at baseline

Of the 25,367 participants we analyzed, the mean age was 50.25 ± 18.08. The mean MLR was 0.31 ± 0.14, and the prevalence of PCa was 3.1%. This study found that the higher MLR tertile population had a higher prevalence of PCa (*p* < 0.05) than the lower tertile group (Table 1). Age, race, BMI, alcohol use, smoking, diabetes, hypertension, TC, triglycerides, ALT, AST, PIR, total bilirubin, serum uric acid, eosinophil percentage, basophils percentage, NLR, PLR, SII, SIRI, and AISI were shown to differ significantly between MLR tertiles (all *p* < 0.05).

**TABLE 1:** Baseline characteristics according to MLR tertiles.

NLR	Overall	Tertile 1	Tertile 2	Tertile 3	*p-*value
		(0.09–1.65)	(1.65–2.25)	(2.25–24.60)	
N	25,367	8,117	8,521	8,729	
MLR	0.31 ± 0.14	0.19 ± 0.04	0.28 ± 0.02	0.45 ± 0.15	<0.001
NLR	2.25 ± 1.27	1.62 ± 0.76	2.03 ± 0.81	3.04 ± 1.58	<0.001
PLR	125.78 ± 52.90	103.00 ± 36.58	118.22 ± 37.80	154.35 ± 64.32	<0.001
SII	533.59 ± 394.90	390.80 ± 250.45	489.32 ± 380.92	709.57 ± 448.77	<0.001
SIRI	1.34 ± 1.01	0.73 ± 0.33	1.16 ± 0.50	2.10 ± 1.30	<0.001
AISI	324.46 ± 316.05	178.02 ± 107.64	284.64 ± 284.31	499.52 ± 385.33	<0.001
Age, years					<0.001
20–40	8,728 (34.41%)	3,494 (43.05%)	3,170 (37.20%)	2064 (23.65%)	
41–60	8,276 (32.63%)	2,940 (36.22%)	2,901 (34.05%)	2,435 (27.90%)	
> 60	8,363 (32.97%)	1,683 (20.73%)	2,450 (28.75%)	4,230 (48.46%)	
Race, n (%)					<0.001
Mexican American	4,326 (17.05%)	1,620 (19.96%)	1,580 (18.54%)	1,126 (12.90%)	
Other Hispanic	2000 (7.88%)	666 (8.21%)	753 (8.84%)	581 (6.66%)	
Non-Hispanic White	11,377 (44.85%)	2,624 (32.33%)	3,775 (44.30%)	4,978 (57.03%)	
Non-Hispanic Black	5,266 (20.76%)	2,204 (27.15%)	1,601 (18.79%)	1,461 (16.74%)	
Other Races	2,398 (9.45%)	1,003 (12.36%)	812 (9.53%)	583 (6.68%)	
Education level, n (%)					0.088
Less than high school	6,963 (27.48%)	2,278 (28.08%)	2,384 (28.01%)	2,301 (26.41%)	
High school or GED	6,068 (23.95%)	1913 (23.58%)	2041 (23.98%)	2,114 (24.26%)	
Above high school	12,306 (48.57%)	3,921 (48.34%)	4,086 (48.01%)	4,299 (49.33%)	
BMI, n (%)					0.089
Normal weight	7,014 (28.17%)	2,254 (28.16%)	2,303 (27.43%)	2,457 (28.92%)	
Overweight	9,656 (38.79%)	3,092 (38.63%)	3,246 (38.67%)	3,318 (39.05%)	
Obese	8,226 (33.04%)	2,658 (33.21%)	2,846 (33.90%)	2,722 (32.03%)	
Smoking status, n (%)					<0.001
≥100 cigarettes lifetime	4,258 (52.48%)	4,677 (54.94%)	5,109 (58.61%)	4,258 (52.48%)	
<100 cigarettes lifetime	3,855 (47.52%)	6,845 (84.33%)	7,103 (83.36%)	6,952 (79.64%)	
PIR, n (%)					<0.001
Low income	2,402 (32.58%)	2,537 (32.62%)	2,686 (33.74%)	2,402 (32.58%)	
Medium income	2,775 (37.64%)	2,949 (37.92%)	3,152 (39.60%)	2,775 (37.64%)	
High income	2,195 (29.77%)	2,291 (29.46%)	2,122 (26.66%)	2,195 (29.77%)	
Alcohol consumption, n (%)	17,170 (78.26%)	5,546 (80.09%)	5,872 (79.37%)	5,752 (75.51%)	<0.001
Hypertension, n (%)	14,569 (57.43%)	4,216 (51.94%)	4,663 (54.72%)	5,690 (65.19%)	<0.001
Diabetes, n (%)	4,467 (17.61%)	1,272 (15.67%)	1,418 (16.64%)	1777 (20.36%)	<0.001
Eosinophil percentage, %	3.09 ± 2.28	3.02 ± 2.25	3.10 ± 2.30	3.15 ± 2.29	0.001
Basophils percentage, %	0.71 ± 0.47	0.73 ± 0.52	0.71 ± 0.44	0.70 ± 0.45	<0.001
TC, mg/dL	191.24 ± 42.06	195.64 ± 42.53	193.13 ± 41.35	185.34 ± 41.64	<0.001
ALT, U/L	29.10 ± 27.19	29.66 ± 19.12	29.51 ± 24.65	28.17 ± 34.84	<0.001
AST, U/L	27.20 ± 21.49	26.87 ± 15.39	26.90 ± 17.19	27.80 ± 28.85	0.006
Triglyceride, mg/dL	163.65 ± 139.56	179.26 ± 162.44	166.24 ± 144.80	146.64 ± 105.37	<0.001
Total bilirubin, mg/dL	0.73 ± 0.34	0.72 ± 0.33	0.72 ± 0.32	0.74 ± 0.36	<0.001
Serum uric acid, mg/dL	6.05 ± 1.33	5.99 ± 1.27	6.03 ± 1.30	6.12 ± 1.42	<0.001
Serum total calcium, mg/dL	9.43 ± 0.37	9.45 ± 0.36	9.44 ± 0.36	9.40 ± 0.38	<0.001
PCa, n (%)	787 (3.10%)	137 (1.69%)	179 (2.10%)	471 (5.40%)	<0.001

MLR, monocyte-to-lymphocyte ratio; NLR, neutrophil-to-lymphocyte ratio; PLR, platelet-to-lymphocyte ratio; SII, systemic immune-inflammation index; SIRI, system inflammation response index; AISI, aggregate index of systemic inflammation; GED, general educational development; BMI, body mass index; PIR, family income to poverty ratio; TC, total cholesterol; ALT: alanine aminotransferase; AST: aspartate aminotransferase; PCa, prostate cancer.

### 3.2 Association between MLR and PCa

Table 2 displays the associations between PCa and MLR and additional inflammatory biomarkers. Based on the findings of our investigation, MLR, NLR, PLR, SII, and AISI were positively correlated with PCa in Models 1 and 2. After completely adjusting for covariates (Model 3), MLR, NLR, PLR, SII, and AISI remained positively correlated with PCa (MLR: OR = 2.28; 95% CI: 1.44, 3.62; NLR: OR = 1.10; 95% CI: 1.04, 1.16; PLR: OR = 1.00; 95% CI: 1.00, 1.01; SII: OR = 1.00; 95% CI: 1.00, 1.01; AISI: OR = 1.00; 95% CI: 1.00, 1.01). MLR and other inflammatory biomarkers were converted from continuous to categorical variables by performing sensitivity analyses. The prevalence of PCa was higher in Model 3 among participants in the highest tertile of MLR and PLR compared with those in the lowest tertile (all *p* for trend <0.05). Whereas, those in the higher tertile of NLR, SII, and AISI had a higher prevalence of PCa compared with the lower tertile (all *p* for trend <0.05).

**TABLE 2:** Association between MLR and other inflammatory markers with PCa.

Index	Continuous or categories	Model 1^c^		Model 2^d^		Model 3^e^	
		OR^a^ (95%CI^b^)	*p-*value	OR (95%CI)	*p-*value	OR (95%CI)	*p-*value
MLR	Continuous variable	11.65 (8.38, 16.20)	<0.0001	1.84 (1.25, 2.70)	0.0019	2.28 (1.44, 3.62)	0.0005
	Tertile 1	Reference		Reference		Reference	
	Tertile 2	1.25 (0.99, 1.56)	0.0516	0.98 (0.78, 1.24)	0.8750	0.90 (0.67, 1.19)	0.4484
	Tertile 3	3.32 (2.74, 4.03)	<0.0001	1.35 (1.09, 1.66)	0.0057	1.35 (1.05, 1.74)	0.0213
	*P* for trend	<0.0001		0.0004		0.0012	
NLR	Continuous variable	1.20 (1.16, 1.25)	<0.0001	1.06 (1.02, 1.11)	0.0080	1.10 (1.04, 1.16)	0.0004
	Tertile 1	Reference		Reference		Reference	
	Tertile 2	1.44 (1.18, 1.76)	0.0004	1.30 (1.05, 1.61)	0.0164	1.34 (1.02, 1.74)	0.0323
	Tertile 3	2.47 (2.05, 2.97)	<0.0001	1.38 (1.13, 1.70)	0.0018	1.60 (1.24, 2.06)	0.0003
	*P* for trend	<0.0001		0.0047		0.0004	
PLR	Continuous variable	1.01 (1.00, 1.01)	<0.0001	1.00 (1.00, 1.01)	<0.0001	1.00 (1.00, 1.01)	<0.0001
	Tertile 1	Reference		Reference		Reference	
	Tertile 2	1.16 (0.95, 1.41)	0.1477	1.16 (0.94, 1.43)	0.1636	1.09 (0.85, 1.41)	0.5057
	Tertile 3	2.14 (1.79, 2.55)	<0.0001	1.62 (1.34, 1.95)	<0.0001	1.76 (1.40, 2.21)	<0.0001
	*P* for trend	<0.0001		<0.0001		<0.0001	
SII	Continuous variable	1.00 (1.00, 1.01)	<0.0001	1.00 (1.00, 1.01)	0.0076	1.00 (1.00, 1.01)	0.0013
	Tertile 1	Reference		Reference		Reference	
	Tertile 2	1.27 (1.05, 1.53)	0.0143	1.32 (1.08, 1.62)	0.0062	1.42 (1.11, 1.83)	0.0057
	Tertile 3	1.79 (1.50, 2.14)	<0.0001	1.48 (1.22, 1.79)	<0.0001	1.80 (1.41, 2.28)	<0.0001
	*P* for trend	<0.0001		<0.0001		<0.0001	
SIRI	Continuous variable	1.21 (1.16, 1.27)	<0.0001	1.02 (0.97, 1.08)	0.4300	1.06 (0.99, 1.13)	0.1074
	Tertile 1	Reference		Reference		Reference	
	Tertile 2	1.25 (1.03, 1.53)	0.0251	1.05 (0.85, 1.30)	0.6571	1.18 (0.91, 1.53)	0.2152
	Tertile 3	2.11 (1.77, 2.53)	<0.0001	1.12 (0.91, 1.37)	0.2760	1.22 (0.95, 1.57)	0.1227
	*P* for trend	<0.0001		0.2575		0.1818	
AISI	Continuous variable	1.00 (1.00, 1.01)	<0.0001	1.00 (1.00, 1.01)	<0.0001	1.00 (1.00, 1.01)	0.0202
	Tertile 1	Reference		Reference		Reference	
	Tertile 2	1.28 (1.06, 1.54)	0.0094	1.24 (1.01, 1.51)	0.0380	1.44 (1.13, 1.85)	0.0034
	Tertile 3	1.65 (1.38, 1.97)	<0.0001	1.21 (0.99, 1.47)	0.0575	1.38 (1.08, 1.76)	0.0089
	*P* for trend	<0.0001		0.1401		0.0453	

In sensitivity analysis, MLR, NLR, PLR, SII, SIRI, and AISI, were converted from continuous variables to categorical variables (tertiles).

^a^ OR: odd ratio.

^b^ 95% CI: 95% confidence interval.

^c^ Model 1: No covariates were adjusted.

^d^ Model 2: Adjusted for age, education level, and race.

^e^ Model 3: Adjusted for age, race, education level, serum uric acid, serum total calcium, TC, triglycerides, total bilirubin; BMI, eosinophil percentage, basophils percentage, smoking status, alcohol consumption, AST, ALT, PIR, diabetes, and hypertension.

**FIGURE 2:**
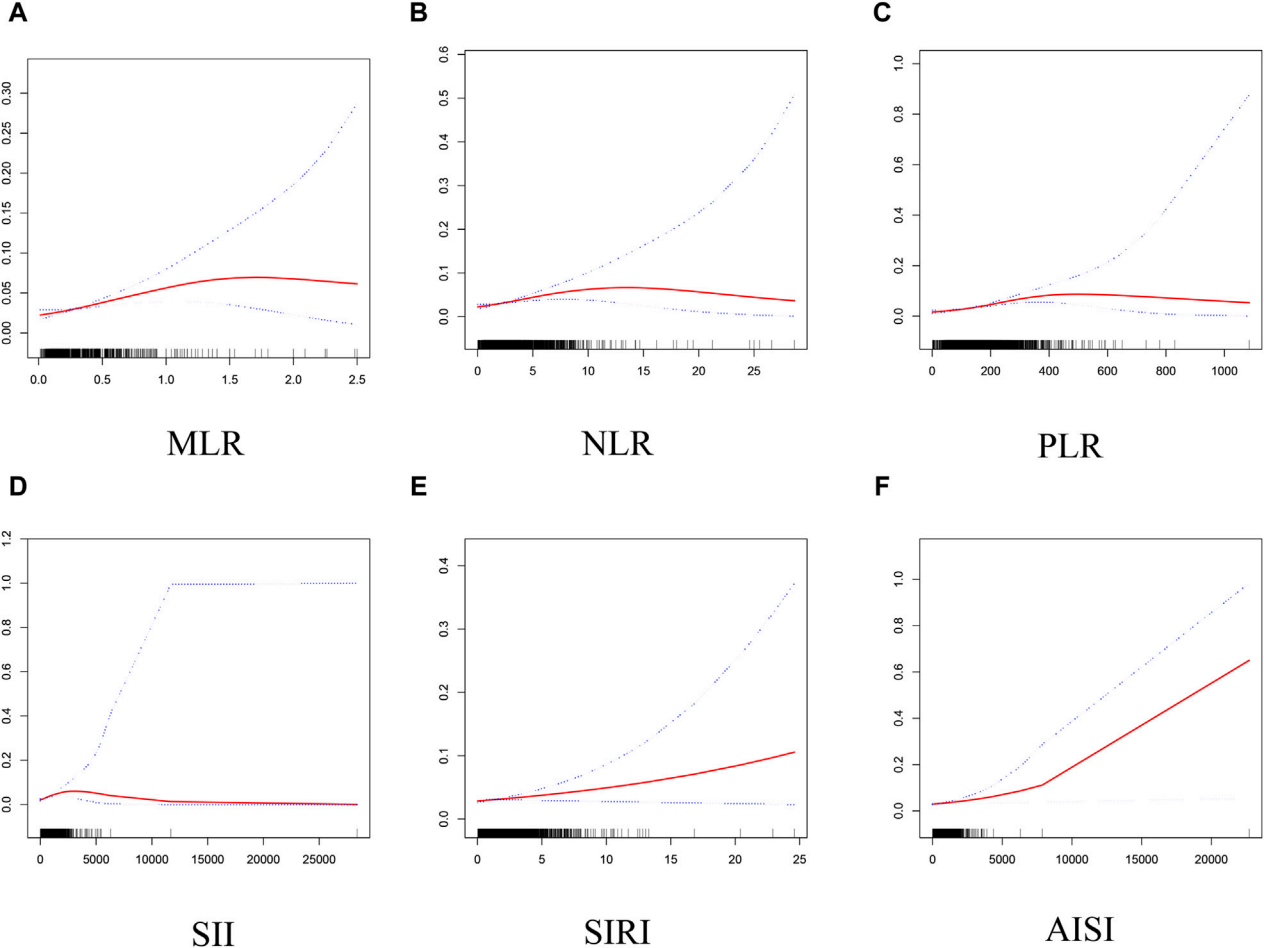
Smooth curve fitting for MLR and other inflammatory markers with PCa. **(A)** MLR and PCa; **(B)** NLR and PCa; **(C)** PLR and PCa; **(D)** SII and PCa; **(E)** SIRI and PCa; **(F)** AISI and PCa.

Based on GAM and smooth curve fitting, the associations between NLR, PLR, SII, and AISI with PCa were nonlinear (Figure 2). Table 3 shows that their breakpoints (K) were 4.32, 215, 822.68, and 257.6, respectively (all log-likelihood ratio test *p* values <0.05). The relationship between MLR and PCa was not shown to be nonlinear.

**TABLE 3:** Threshold effect analysis of MLR and other inflammatory markers on PCa using a two-piecewise linear regression model in Model 3.

	MLR	NLR	PLR	SII	SIRI	AISI
Standard linear model						
OR^a^ (95%CI^b^)	2.28 (1.44, 3.62)	1.10 (1.04, 1.16)	1.00 (1.00, 1.01)	1.00 (1.00, 1.01)	1.06 (0.99, 1.13)	1.00 (1.00, 1.01)
*p-*value	0.0005	0.0004	<0.0001	0.0013	0.1074	0.0202
Fitting by two-piecewise linear model						
Breakpoint (K)	0.5	4.32	215	822.68	1.36	257.6
OR1 (<K)	4.77 (1.89, 12.02)	1.20 (1.09, 1.32)	1.01 (1.00, 1.01)	1.00 (1.00, 1.01)	1.48 (1.06, 2.07)	1.00 (1.00, 1.01)
	0.0009	0.0002	<0.0001	<0.0001	0.0199	0.0073
OR2 (>K)	1.38 (0.65, 2.92)	1.02 (0.92, 1.12)	1.00 (0.99, 1.00)	1.00 (0.99, 1.00)	1.01 (0.93, 1.11)	1.00 (0.99, 1.00)
	0.3972	0.7072	0.2014	0.8444	0.7499	0.2863
OR2/OR1	0.29 (0.07, 1.12)	0.85 (0.72, 0.99)	0.98 (0.98, 0.99)	0.99 (0.98, 0.99)	0.68 (0.47, 0.99)	1.00 (1.00, 1.01)
	0.0734	0.0383	0.0267	0.0008	0.0419	0.0134
Logarithmic likelihood ratio test *p*-value	0.069	0.032	0.024	<0.001	0.039	0.011

Adjusted for age, race, education level, serum uric acid, serum total calcium, TC, triglycerides, total bilirubin; BMI, eosinophil percentage, basophils percentage, smoking status, alcohol consumption, AST, ALT, PIR, diabetes, and hypertension.

^a^ OR: odd ratio.

^b^ 95% CI: 95% confidence interval.

### 3.3 Subgroup analysis

**FIGURE 3:**
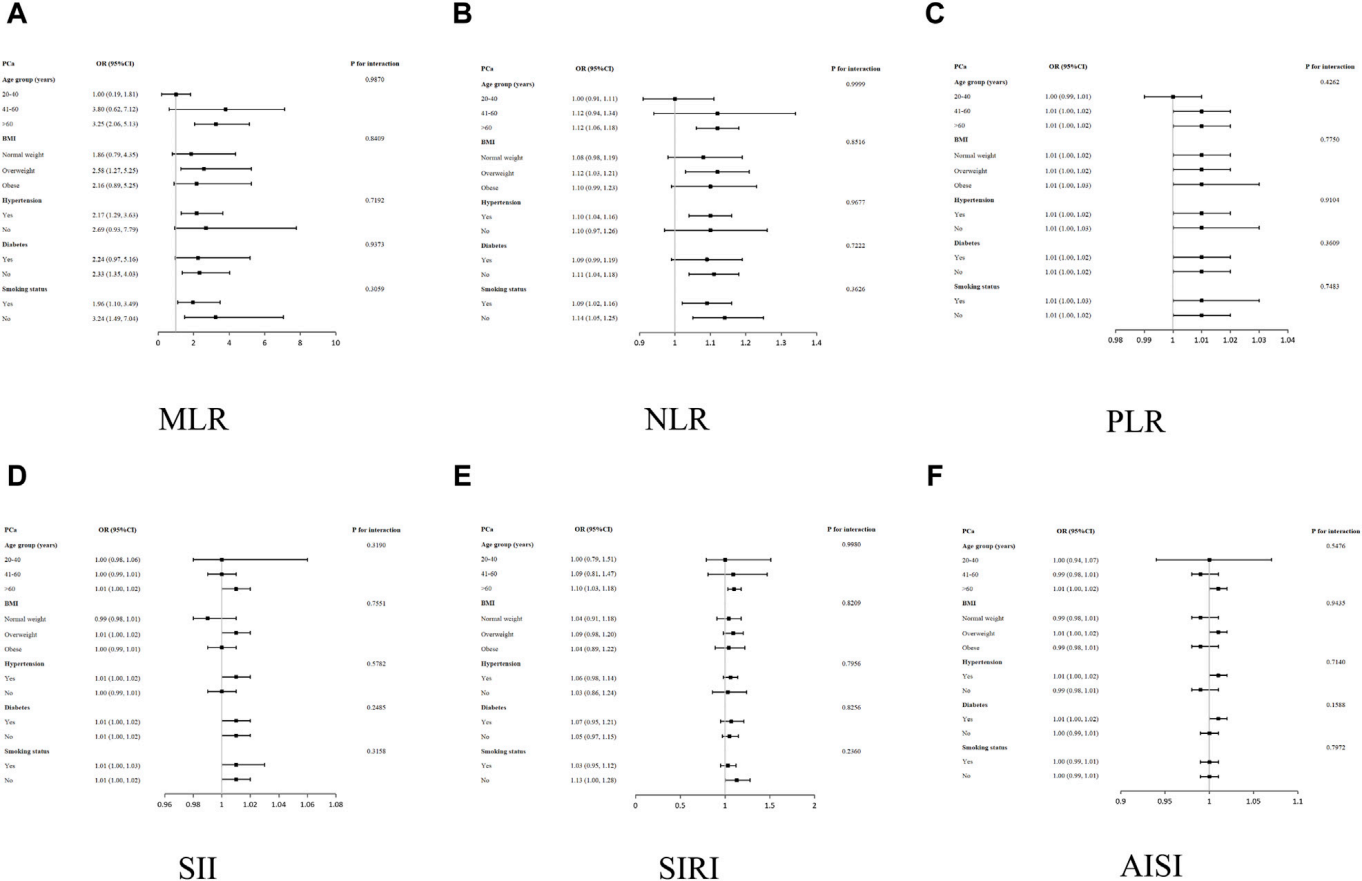
Subgroup analysis for the associations of NLR and other inflammatory markers with PCa. **(A)** MLR and PCa; **(B)** NLR and PCa; **(C)** PLR and PCa; **(D)** SII and PCa; **(E)** SIRI and PCa; **(F)** AISI and PCa.

In all groupings of the smoking status, MLR was correlated with PCa positively. However, no significant correlation was found between MLR and PCa in the age group of 20–60 years, normal weight and obese, diabetic and non-hypertensive populations. Age, hypertension, BMI, diabetes, and smoking status did not substantially alter the association between MLR and PCa in any stratum, according to the interaction tests (Figure 3). Similarly, the relationship between inflammatory biomarkers (NLR, PLR, SII, SIRI, AISI) and PCa remained stable in each population (all *p* for interaction >0.05).

### 3.4 ROC analysis

**FIGURE 4:**
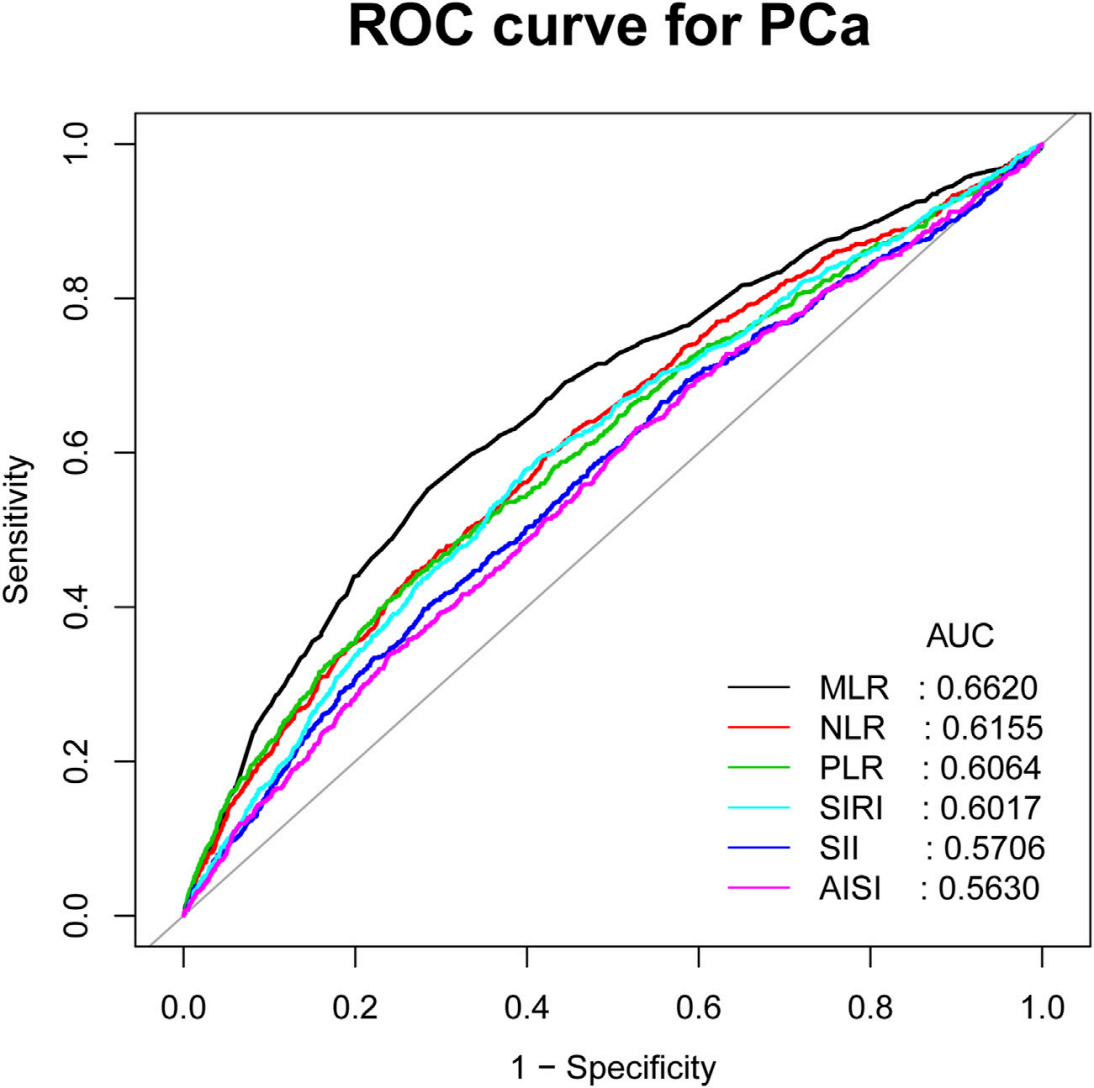
ROC curves and the AUC values of the six inflammatory markers (NLR, MLR, PLR, SII, SIRI, and AISI) in diagnosing PCa.

Figure 4 shows the comparison of AUC values between MLR and other inflammatory biomarkers (NLR, PLR, SII, SIRI, and AISI) to establish their predictive power for PCa. Our study results showed that MLR had a higher AUC value than the other five inflammatory biomarkers for predicting PCa (AUC: 0.6620, 95% CI: 0.6417–0.6823). The differences in AUC values between them were statistically significant (all *p* < 0.05) (Table 4). These findings showed that MLR outperformed other inflammatory biomarkers (NLR, PLR, SII, SIRI, and AISI) and had a better discrimination ability and accuracy for predicting PCa.

**TABLE 4:** Comparison of AUC values between MLR and other inflammatory markers.

Test	AUC^a^	95%CI^b^ low	95%CI upp	Best threshold	Specificity	Sensitivity	*P* For different in AUC
MLR	0.6620	0.6417	0.6823	0.1331	0.7156	0.5527	Reference
NLR	0.6155	0.5948	0.6362	0.0899	0.7325	0.4447	<0.0001
PLR	0.6064	0.5850	0.6279	0.1335	0.7424	0.4282	<0.0001
SII	0.5706	0.5494	0.5917	0.0215	0.7202	0.3977	<0.0001
SIRI	0.6017	0.5 810	0.6223	0.0508	0.6031	0.5769	<0.0001
AISI	0.5630	0.5421	0.5839	0.0105	0.4723	0.6315	<0.0001

^a^ AUC: area under the curve.

^b^ 95% CI: 95% confidence interval.

### 3.5 Association between MLR and PSA

The relationships between PSA and inflammatory indicators were explored as well. In Model 3, PSA levels increase with increasing MLR, NLR, and PLR levels (Supplementary Table S1). We discovered a saturation effect between NLR and PSA, with a breakpoint around 4.55 (Supplementary Table S2), in accordance with GAM and smooth curve fitting. Nevertheless, a nonlinear association between MLR and PSA was not discovered (Supplementary Figure S1).

## 4 Discussion

We discovered a positive connection between MLR and PCa prevalence in this cross-sectional study involving 25,367 adults. Moreover, higher levels of NLR, PLR, SII, and AISI were associated with higher PCa prevalence. The link between MLR and other inflammatory biomarkers (NLR, PLR, SII, SIRI, and AISI) with PCa did not significantly differ throughout the populations, according to subgroup analysis and interaction tests. MLR may be a more accurate inflammatory biomarker than NLR, PLR, SII, SIRI, and AISI in predicting PCa, according to ROC analysis. In conclusion, a high level of MLR should be paid attention to in the assessment of PCa in American adults.

Previous studies with different epidemiologic methods and target populations have reported the association between MLR and PCa. A retrospective study involving 319 Chinese patients found that MLR levels were shown to be considerably higher in PCa patients (Zhu et al., 2023). Another cohort study by Xu et al. in a Chinese population demonstrated that MLR may be a good predictor of PCa(Xu et al., 2021). Two other studies revealed that MLR is an important predictor of PCa diagnosis (Hou et al., 2020; Zhou et al., 2021). However, some studies have also shown the limitations of MLR. A retrospective study by Pawel et al. (Rajwa et al., 2021) concluded that MLR was not useful in the diagnosis of PCa. Racial differences in the association between MLR and PCa may also exist, as shown in a racially diverse study with a longer follow-up (Rundle et al., 2021). Our study has some advantages over previous ones. First, previous findings of the association between MLR and PCa have been inconsistent. Our study further confirmed the reliability of the association between MLR and PCa. This study examined the association between MLR and PCa in the broader US population for the first time. Furthermore, we concurrently explored the association of other inflammatory biomarkers and Pca and assessed their predictive ability through ROC analysis. This is a fundamental difference from previous studies.

The potential diagnostic utility of other kinds of systemic inflammatory biomarkers in PCa has been investigated in an increasing number of studies. Luo et al. demonstrated that SII was positively correlated with an increased risk of PCa (Luo et al., 2023). A population-based cohort study found that SII could provide better PCa screening methods before biopsy (Wang et al., 2021). Our study also agreed with this view, as elevated levels of SII were associated with a higher prevalence of PCa. Furthermore, we discovered that the prevalence of PCa rose by 10% for each unit rise in NLR. This may be connected to neutrophils stimulating the development and spread of tumors by immune suppression, angiogenesis, and metastasis, according to earlier research (Bekes et al., 2011; Coffelt et al., 2015; Masuda et al., 2021). We further discovered the threshold effect and saturation effect of NLR on PCa. The breakpoint was 4.32. The prevalence of PCa increases with increasing NLR levels on the left side of the breakpoint. There was no correlation discovered between the two on the right side.

We also investigated the positive association between PCa with AISI and PLR, as was done in earlier research (Adhyatma and Warli, 2019; Xie et al., 2023). We did not observe a correlation between SIRI and PCa, which is in contrast to previous study results (Bailey-Whyte et al., 2023). This may be related to different populations and regions. Further in-depth prospective studies are needed to verify our conclusions.

The main finding of this study was the positive correlation between MLR and PCa. In other words, the prevalence of PCa increased by 1.28 times with every unit increase in MLR. This is similar to previous studies (Hou et al., 2020; Zhu et al., 2023). Therefore, the potential risk of PCa should not be neglected by American adults with high MLR levels. The reason for the significant association between MLR and PCa may be that MLR can combine the effects of monocytes and lymphocytes. Monocytes in the bloodstream can produce reactive oxygen, reactive nitrogen, and other cytokines, and inhibit lymphocyte activation, causing DNA mutations and tumor progression (Pollard, 2004). Lymphocytes can cause tumor cell apoptosis and reduce tumor growth by cytotoxic activity, performing an anti-cancer immunity function in the blood circulation and tumor microenvironment (Augier et al., 2010; Tiainen et al., 2021). Therefore, high MLR can signify the low anti-tumor immunity of the body, which is linked to the increased risk of cancer, as monocyte increase usually coincides with relative lymphocyte decrease.

Previous studies have also explored the advantages of MLR in predicting other diseases. It was reported that MLR might be a better predictor than NLR in predicting the prognosis of patients with acute ischemic stroke (AIS) (Wang C. J. et al., 2022). MLR was also confirmed to be superior to NLR in predicting acute kidney injury and in-hospital mortality after acute hemorrhagic stroke (Jiang et al., 2022). Our study also supports this view, ROC analysis showed that MLR might be better for predicting PCa than other inflammatory biomarkers (NLR, PLR, SII, SIRI, and AISI). In summary, MLR, as a noninvasive and low-cost method, has a good clinical application prospect in predicting PCa risk.

When it comes to PCa diagnosis and treatment, age may play a crucial role. As people age, their immune system function declines along with them, which causes attenuation in immune surveillance and elicits the risk of developing malignancies (Agrawal and Gupta, 2011; Lian et al., 2020). A study found that the older the age, the higher the risk of PCa, and the higher the risk of more aggressive PCa (Godtman et al., 2022). Our previous study confirmed this point, in the population aged >60 years, for every unit increase in MLR, the prevalence of PCa increased by 2.25 times. And in the population aged 20–60 years, there was no significant association between the two. Moreover, according to the interaction tests, age, BMI, hypertension, diabetes and smoking status did not affect the relationship significantly between MLR and PCa in any stratum. This result further verified the importance of MLR in predicting PCa risk for the American adult population.

Studies have found that PSA is one of the important risk factors for predicting PCa (Xu et al., 2021). Our study showed that the increase in MLR level was significantly correlated with the increase in PSA level. High Gleason score (GS) and benign prostatic hyperplasia (BPH) were independent predictors of PCa. MLR was also proven to be significantly positively correlated with high GS and BPH (Hayashi et al., 2017; Chen and Feng, 2023). Therefore, we cannot ignore the importance of MLR in identifying PCa.

The mechanism between inflammation and PCa has received extensive attention (Gobel et al., 2021). Via a variety of chemicals, inflammatory cells contribute to DNA mutations, apoptosis resistance, cell cycle regulation, cancer invasion and metastasis (Hanahan and Weinberg, 2011). Lesions such as proliferative inflammatory atrophy, which is brought on by a series of inflammatory responses, may be possible precursors of PCa (De Marzo et al., 2007; Davidsson et al., 2011). Some inflammatory cytokines are linked to prostate cancer risk, such as interleukin-8 (IL-8), a key inflammatory mediator, secreted by various cells, such as lymphocytes, monocytes, endothelial cells, etc. (Hebert and Baker, 1993; Sun et al., 2007). Furthermore, it has been noted that IL-8 expression is connected to tumor characteristics like angiogenesis and lymph node metastasis in human prostate cancer cells (Kim et al., 2001). Through its interaction with the epidermal growth factor receptor (EGFR) signaling pathway, IL-8 may contribute to the progression of prostate cancer (Singh and Lokeshwar, 2011).

Our research holds its own strength. We used data from NHANES, which has a representative and large sample size. To get more trustworthy results, we also adjusted for confounding factors. We also clarified the stronger correlation between MLR and PCa. MLR, as a noninvasive and easily accessible biomarker, was a promising tool for predicting patients with PCa. However, there are still several restrictions on our research. For example, we were unable to determine causality due to the cross-sectional study design. Additionally, The collected sample data were derived from a single blood test and might not be a reliable representation of the inflammatory and immune status over an extended period of time. We may have overlooked some more potential confounding factors that could have affected our results, even though we controlled for some of them. We adjusted for some of them. Finally, we don't have internal and external validation cohorts to verify the reliability of the results.

## 5 Conclusion

MLR might be better than other inflammatory biomarkers (NLR, PLR, SII, SIRI, and AISI) in predicting PCa. Adult Americans with increased MLR, NLR, PLR, SII, and AISI levels ought to be conscious of their possible PCa risk. To validate the importance of MLR in the diagnosis and treatment of PCa, more large-scale prospective studies are still needed.

## Data Availability

Publicly available datasets were analyzed in this study. This data can be found here: https://www.cdc.gov/nchs/nhanes/.

## Supplementary material

SUPPLEMENTARY FIGURE S1 Smooth curve fitting for MLR and other inflammatory markers with PSA. **(A)** MLR and PSA; **(B)** NLR and PSA; **(C)** PLR and PSA; **(D)** SII and PSA; **(E)** SIRI and PSA; **(F)** AISI and PSA.
