# Supplementary material for: Association between monocyte-to-lymphocyte ratio and prostate cancer in the U.S. population: a population-based study
Source: Front Cell Dev Biol. 2024 Apr 5;12:1372731. doi: 10.3389/fcell.2024.1372731 (PMC11026607; doi:10.3389/fcell.2024.1372731)
Supplement: Supplementary file 1 [file Table1.DOCX]

**Supplementary Table S1** Association between MLR and other inflammatory markers with PSA.

| **Index** | **Continuous or categories** | Model 1^3^ | | Model 2^4^ | | [Model 3](https://www.ncbi.nlm.nih.gov/pmc/articles/PMC8987107/table/T2/?report=objectonly#t2fna)^5^ | |
| --- | --- | --- | --- | --- | --- | --- | --- |
|  |  | β^1^ (95%CI^2^) | *P-* value | β (95%CI) | *P-* value | β (95%CI) | *P-* value |
| **MLR** | Continuous variable | 2.42 (1.78, 3.06) | <0.0001 | 0.85 (0.19, 1.51) | 0.0122 | 0.80 (0.20, 1.41) | 0.0094 |
|  | Tertile 1 | Reference |  | Reference |  | Reference |  |
|  | Tertile 2 | 0.19 (-0.05, 0.42) | 0.1201 | 0.07 (-0.17, 0.30) | 0.5755 | 0.06 (-0.14, 0.26) | 0.5759 |
|  | Tertile 3 | 0.78 (0.56, 1.00) | <0.0001 | 0.28 (0.05, 0.51) | 0.0163 | 0.18 (-0.03, 0.38) | 0.0882 |
|  | *P* for trend | <0.0001 |  | 0.0128 |  | 0.0828 |  |
| **NLR** | Continuous variable | 0.21 (0.14, 0.28) | <0.0001 | 0.10 (0.03, 0.17) | 0.0069 | 0.08 (0.02, 0.14) | 0.0107 |
|  | Tertile 1 | Reference |  | Reference |  | Reference |  |
|  | Tertile 2 | 0.30 (0.07, 0.52) | 0.0111 | 0.25 (0.03, 0.48) | 0.0275 | 0.19 (-0.01, 0.39) | 0.0547 |
|  | Tertile 3 | 0.74 (0.51, 0.96) | <0.0001 | 0.42 (0.19, 0.65) | 0.0004 | 0.30 (0.09, 0.50) | 0.0047 |
|  | *P* for trend | <0.0001 |  | 0.0006 |  | 0.0063 |  |
| **PLR** | Continuous variable | 0.01 (0.01, 0.01) | <0.0001 | 0.01 (0.01, 0.01) | 0.0001 | 0.01 (0.01, 0.01) | 0.0006 |
|  | Tertile 1 | Reference |  | Reference |  | Reference |  |
|  | Tertile 2 | 0.23 (0.01, 0.46) | 0.0458 | 0.28 (0.06, 0.50) | 0.0127 | 0.09 (-0.10, 0.28) | 0.3511 |
|  | Tertile 3 | 0.52 (0.29, 0.75) | <0.0001 | 0.44 (0.21, 0.66) | 0.0001 | 0.35 (0.15, 0.54) | 0.0007 |
|  | *P* for trend | <0.0001 |  | 0.0002 |  | 0.0005 |  |
| **SII** | Continuous variable | 0.01 (0.01, 0.01) | 0.0009 | 0.01 (0.01, 0.01) | 0.0226 | 0.01 (-0.01, 0.01) | 0.0902 |
|  | Tertile 1 | Reference |  | Reference |  | Reference |  |
|  | Tertile 2 | 0.11 (-0.12, 0.33) | 0.3562 | 0.17 (-0.06, 0.39) | 0.1465 | 0.04 (-0.15, 0.24) | 0.6697 |
|  | Tertile 3 | 0.61 (0.38, 0.84) | <0.0001 | 0.53 (0.31, 0.76) | <0.0001 | 0.33 (0.13, 0.53) | 0.0013 |
|  | *P* for trend | <0.0001 |  | <0.0001 |  | 0.0007 |  |
| **SIRI** | Continuous variable | 0.27 (0.18, 0.36) | <0.0001 | 0.11 (0.02, 0.20) | 0.0182 | 0.08 (-0.01, 0.16) | 0.0580 |
|  | Tertile 1 | Reference |  | Reference |  | Reference |  |
|  | Tertile 2 | 0.20 (-0.03, 0.43) | 0.0874 | 0.10 (-0.12, 0.33) | 0.10 (-0.12, 0.33) | 0.04 (-0.16, 0.24) | 0.6770 |
|  | Tertile 3 | 0.73 (0.50, 0.96) | <0.0001 | 0.36 (0.13, 0.60) | 0.0026 | 0.25 (0.04, 0.46) | 0.0180 |
|  | *P* for trend | <0.0001 |  | 0.0018 |  | 0.0121 |  |
| **AISI** | Continuous variable | 0.01 (0.01, 0.01) | 0.0007 | 0.01 (-0.01, 0.01) | 0.0543 | 0.01 (-0.01, 0.01) | 0.2324 |
|  | Tertile 1 | Reference |  | Reference |  | Reference |  |
|  | Tertile 2 | 0.20 (-0.02, 0.43) | 0.0805 | 0.19 (-0.03, 0.42) | 0.0960 | 0.10 (-0.10, 0.29) | 0.3266 |
|  | Tertile 3 | 0.57 (0.34, 0.80) | <0.0001 | 0.42 (0.19, 0.65) | 0.0004 | 0.30 (0.09, 0.50) | 0.0045 |
|  | *P* for trend | <0.0001 |  | 0.0004 |  | 0.0037 |  |

In sensitivity analysis, MLR, NLR, PLR, SII, SIRI, and AISI were converted from continuous variables to categorical variables (tertiles).

^1^β: effect size.

^2^95% CI: 95% confidence interval.

^3^Model 1: No covariates were adjusted.

^4^Model 2: Adjusted for age, education level, and race.

^5^Model 3: Adjusted for age, race, education level, serum uric acid, serum total calcium, TC, triglycerides, total bilirubin, BMI, eosinophil percentage, basophils percentage, smoking status, alcohol consumption, AST, ALT, PIR, diabetes, and hypertension.
