# Supplementary figures and images for: Association between monocyte-to-lymphocyte ratio and prostate cancer in the U.S. population: a population-based study
Source: Front Cell Dev Biol. 2024 Apr 5;12:1372731. doi: 10.3389/fcell.2024.1372731 (PMC11026607; doi:10.3389/fcell.2024.1372731)

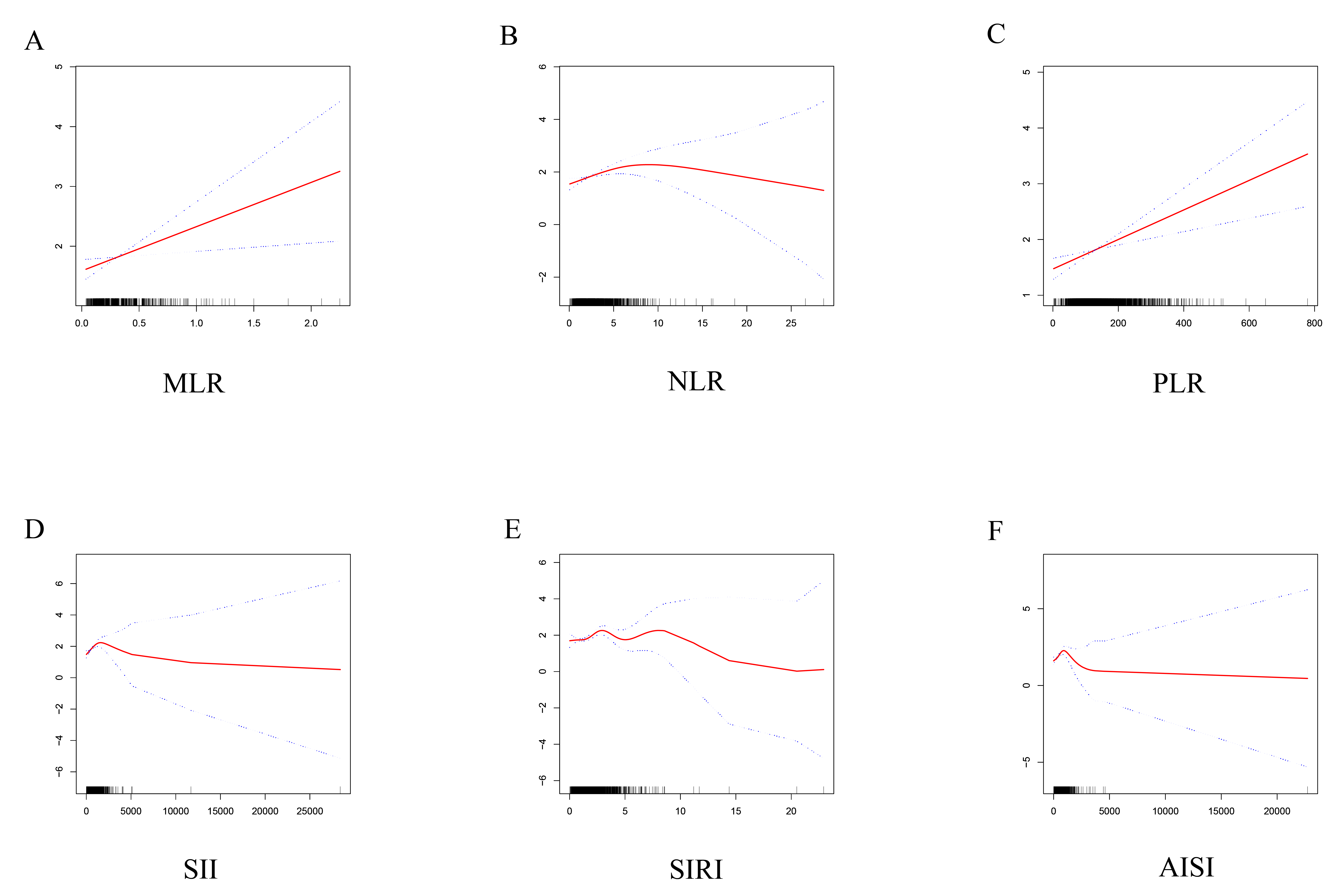

Supplement: Supplementary file 2 [file Image1.TIF]
