# Supplementary material for: Association between monocyte-to-lymphocyte ratio and prostate cancer in the U.S. population: a population-based study
Source: Front Cell Dev Biol. 2024 Apr 5;12:1372731. doi: 10.3389/fcell.2024.1372731 (PMC11026607; doi:10.3389/fcell.2024.1372731)
Supplement: Supplementary file 3 [file Table2.DOCX]

**Supplementary Table S2** Threshold effect analysis of MLR and other inflammatory markers on PSA using a two-piecewise linear regression model in Model 3.

|  | **MLR** | **NLR** | **PLR** | **SII** | **SIRI** | **AISI** |
| --- | --- | --- | --- | --- | --- | --- |
| Standard linear model |  |  |  |  |  |  |
| β^1^ (95%CI^2^) | 0.80 (0.20, 1.41) | 0.08 (0.02, 0.14) | 0.01 (0.01, 0.01) | 0.01 (-0.01, 0.01) | 0.08 (-0.00, 0.16) | 0.01 (-0.01, 0.01) |
| *P-*value | 0.0094 | 0.0107 | 0.0006 | 0.0902 | 0.0580 | 0.2324 |
| **Fitting by two-piecewise linear model** |  |  |  |  |  |  |
| Breakpoint (K) | 0.15 | 4.55 | 160.56 | 1158.95 | 2.93 | 789.75 |
| β1(< K ) | -7.68 (-17.63, 2.27) | 0.16 (0.07, 0.25) | 0.01 (0.01, 0.01) | 0.01 (0.01, 0.01) | 0.24 (0.11, 0.37) | 0.01 (0.01, 0.01) |
|  | 0.1306 | 0.0006 | 0.0046 | <0.0001 | 0.0003 | <0.0001 |
| β2(> K ) | 0.93 (0.31, 1.55) | -0.04 (-0.16, 0.08) | 0.01 (-0.01, 0.01) | -0.01 (-0.01, 0.01) | -0.12 (-0.26, 0.03) | -0.01 (-0.01, 0.01) |
|  | 0.0035 | 0.5042 | 0.3254 | 0.2619 | 0.1205 | 0.1606 |
| β2 / β1 | 8.34 (-1.71, 18.39) | -0.20 (-0.37, -0.03) | -0.01 (-0.01, 0.01) | -0.01 (-0.01, -0.01) | -0.36 (-0.58, -0.13) | -0.01 (-0.01, -0.01) |
|  | 0.0943 | 0.0193 | 0.2557 | <0.0001 | 0.0017 | <0.0001 |
| Logarithmic likelihood ratio test P-value | 0.093 | 0.019 | 0.254 | <0.001 | 0.002 | <0.001 |

Adjusted for age, race, education level, serum uric acid, serum total calcium, TC, triglycerides, total bilirubin, BMI, eosinophil percentage, basophils percentage, smoking status, alcohol consumption, AST, ALT, PIR, diabetes, and hypertension.

^1^β: effect size..

^2^95% CI: 95% confidence interval.
